# Molecular Cloning and Characterization of G Alpha Proteins from the Western Tarnished Plant Bug, *Lygus hesperus*

**DOI:** 10.3390/insects6010054

**Published:** 2014-12-31

**Authors:** J. Joe Hull, Meixian Wang

**Affiliations:** 1 USDA-ARS Arid Land Agricultural Center, Maricopa, AZ 85138, USA; E-Mail: meixian.wang@ars.usda.gov; 2 Key Laboratory of Animal Virology of Ministry of Agriculture, College of Animal Sciences, Zhejiang University, Hangzhou 310058, China

**Keywords:** *Lygus hesperus*, plant bug, heterotrimeric G protein, signal transduction, Gα subunit, gene cloning, expression profile

## Abstract

The Gα subunits of heterotrimeric G proteins play critical roles in the activation of diverse signal transduction cascades. However, the role of these genes in chemosensation remains to be fully elucidated. To initiate a comprehensive survey of signal transduction genes, we used homology-based cloning methods and transcriptome data mining to identity Gα subunits in the western tarnished plant bug (*Lygus hesperus* Knight). Among the nine sequences identified were single variants of the Gαi, Gαo, Gαs, and Gα12 subfamilies and five alternative splice variants of the Gαq subfamily. Sequence alignment and phylogenetic analyses of the putative *L. hesperus* Gα subunits support initial classifications and are consistent with established evolutionary relationships. End-point PCR-based profiling of the transcripts indicated head specific expression for LhGαq4, and largely ubiquitous expression, albeit at varying levels, for the other LhGα transcripts. All subfamilies were amplified from *L. hesperus* chemosensory tissues, suggesting potential roles in olfaction and/or gustation. Immunohistochemical staining of cultured insect cells transiently expressing recombinant His-tagged LhGαi, LhGαs, and LhGαq1 revealed plasma membrane targeting, suggesting the respective sequences encode functional G protein subunits.

## 1. Introduction

Heterotrimeric guanine-nucleotide-binding proteins (G proteins) are molecular switches that mediate many extracellular signaling processes by coupling cell surface receptor activation with the diverse signal transduction effector molecules that drive cellular responses. The heterotrimeric G protein complex is composed of an α-subunit (Gα) that functions in guanine nucleotide binding/hydrolysis and a heterodimer composed of a β and γ subunit (Gβγ). In the absence of receptor stimulation, the three subunits are associated and GDP is bound to Gα. Receptor activation triggers GDP exchange for GTP and dissociation of Gβγ from the Gα-GTP complex. The dissociated Gα and Gβγ subunits are then able to modulate the activity of various downstream effector proteins (ion channels, adenylyl cyclases, phospholipase Cβ, *etc.*). The intrinsic GTPase activity of Gα hydrolyzes GTP to GDP, which promotes reassociation of the heterotrimeric G protein complex and terminates the signal [1,2,3]. Based on this intermediary molecular role, heterotrimeric G proteins play pivotal roles in determining the specificity and duration of the cellular response to extracellular signals.

The Gα subunits form a large multigene family composed of 39–52 kDa proteins that share 35%–95% sequence identity and have been grouped into four subfamilies (Gαs, Gαi/o, Gαq, and Gα12) based on structural and functional similarities [1,2,3]. Gαs subfamily members couple receptors to adenylyl cyclase stimulation (*i.e.*, increases in cAMP), whereas the Gαi/o subfamily has the opposite effect. The Gαq subfamily regulate the activity of phospholipase C β isoforms (*i.e.*, diacylglycerol and inositol triphosphate production) [1,2] and Gα12 has been extensively characterized based on their ability to activate Rho-specific guanine nucleotide exchange factors [4,5].

Chemosensory signaling in many vertebrates and invertebrates relies on canonical G protein-coupled pathways. In insects, however, the role of G proteins in chemosensory transduction has yet to be definitively established [6,7]. Insect olfactory and gustatory receptors have poor homology with canonical G protein-coupled receptors [8], exhibit inverted topologies [9,10,11,12], and are activated through an ionotropic mechanism in which the receptors function as ligand-gated ion channels [13,14,15]. Other studies though have reported a G protein-coupled metabotropic component in olfactory receptor activation [16]. In support of this pathway, Gα subunits are expressed in chemosensory tissues [17,18,19,20,21], G protein dependent effector pathways are activated by odorants [16,22,23,24], and inhibition of G protein activation negatively affects odorant perception [22,25,26,27] as does RNAi-mediated knockdown of Gα subunits [28,29]. In addition, G protein-coupled pathways have been implicated in gustatory receptor activation [30,31,32,33,34,35,36].

The western tarnished plant bug (*Lygus hesperus*) is a polyphagous pest of numerous crops [37,38] that utilizes chemosensory signals to aid in identification of host plants and conspecific mates [39,40,41,42]. Despite the pest status of the *Lygus* spp. complex, transcriptional resources have only recently been developed [43,44,45,46], and our knowledge of chemosensory signal transduction is limited to odorant binding proteins [45,47] and the olfactory receptor co-receptor (Orco) [12] in *L. lineolaris* and *L. hesperus*. Furthermore, while G proteins have been studied in a number of insects with Gα subunits cloned from *Drosophila melanogaster* [17,21,48,49,50], *Anopheles gambiae* [20], *Bombyx mori* [19,51,52], *Manduca sexta* [53], *Locusta migratoria* [54], *Lissorhoptrus oryzophilus* [55], *Helicoverpa assaulta* [56], *Mamestra brassicae* [18], *Bemisia tabaci* [57], and *Oncopeltus fasciatus* [58], little progress has been made on the role of these genes in mediating chemosensory behaviors in plant bugs such as *Lygus*. In this study, we sought to begin to address this lack of knowledge by identifying the molecular sequences and expression profile of Gα subunits in *L. hesperus*. Using homology-based PCR and transcriptome database mining methods, we cloned a group of cDNAs with high sequence homology to each of the Gα subfamilies. In addition, we performed detailed sequence comparisons of the *L. hesperus* transcripts with those from other insects, profiled transcript expression levels, and examined the subcellular localization of a subset of recombinantly expressed *L. hesperus* Gα proteins in cultured insect cells.

## 2. Experimental Section

### 2.1. Insect Rearing

*L. hesperus* were obtained from an in-house stock colony (USDA-ARS Arid Land Agricultural Research Center, Maricopa, AZ, USA) periodically outbred with locally caught conspecifics. The colony is fed an artificial diet packaged in Parafilm M [59,60] and maintained under rearing conditions consisting of 27 °C, 40% humidity and a L14:D10 photoperiod. Experimental nymphs were generated from eggs deposited in oviposition packets and maintained as described previously [61].

### 2.2. Identification and Cloning of L. hesperus Gα Subunits

**Figure 1:**
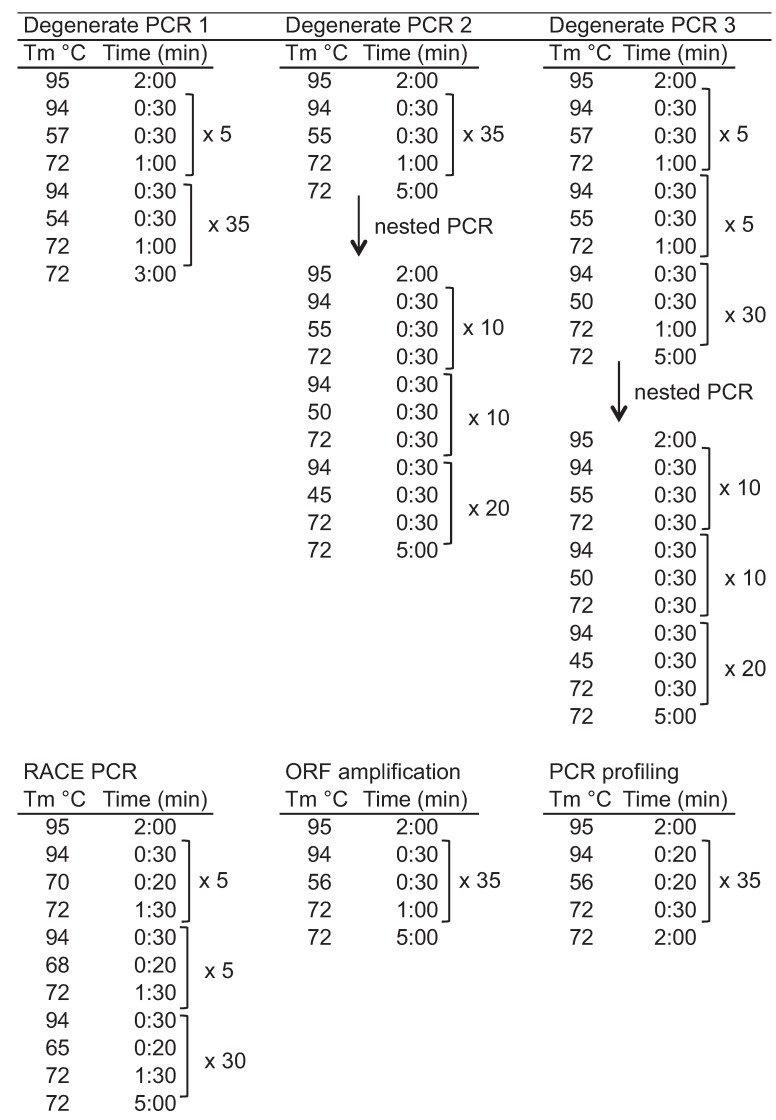
Thermocycler conditions used.

To identify *L. hesperus* Gα subunits (LhGα), we initially utilized a degenerate PCR approach similar to that reported previously in *B. mori* [51,52] using degenerate primers (Table 1) designed to conserved amino acid stretches identified in protein sequence alignments of known insect Gα sequences. Total RNA was isolated from adult *L. hesperus* female heads and bodies using TRI Reagent RNA Isolation Reagent (Sigma-Aldrich, St. Louis, MO, USA) according to the manufacturer’s instructions. Isolated total RNA was quantified based on absorbance at 260 nm using a Take3 multi-volume plate on a Synergy H4 hybrid multi-mode microplate reader (BioTek Instruments, Winooski, VT, USA). First strand cDNA was synthesized from 1 μg of DNase I-treated total RNA in separate Thermoscript or SuperScript III (Life Technologies, Carlsbad, CA, USA) first-strand cDNA synthesis reactions with random hexamers. To minimize primer bias towards particular classes of Gα proteins [62,63], multiple PCR amplifications were performed using ExTaq DNA polymerase (Takara Bio Inc./Clontech, Palo Alto, CA, USA) with 0.7 μL (35 ng) cDNA template and 2.5–3 μL (0.5–0.6 μM) of each primer and varying thermocycler conditions (Figure 1). Nested PCR was performed as above but using a 1-μL aliquot of the previous reaction as the template. PCR products were electrophoresed on 1.7% agarose gels and stained with SYBR Safe (Life Technologies). Amplimers of the expected sizes were gel-excised using an EZNA Gel Extraction kit (Omega Bio-Tek Inc., Norcross, GA, USA), cloned into the pGEM T Easy-TA cloning vector (Promega, Madison, WI, USA) and sequenced at the Arizona State University DNA Core Lab (Tempe, AZ, USA).

The partial fragments amplified above were extended by RACE PCR using templates generated with a SMARTer RACE cDNA Amplification kit (Clontech, Mountain View, CA, USA) and 2 μg DNase I-treated RNA. Amplification was performed using ExTaq with 0.5 μL (50 ng) cDNA, primers corresponding to one of the Universal Primers supplied with the SMARTer RACE cDNA Amplification kit, a gene specific primer (Table 1), and touchdown thermocycler conditions (Figure 1). PCR products were electrophoresed on 1.5% agarose gels with amplimers of the expected sizes gel excised and sequenced. Incorporating the resulting 5' and 3' RACE sequence data with the degenerate PCR derived sequences yielded sufficient data to design gene specific primers encompassing the putative start and stop codons (Table 1). The respective *L. hesperus* Gα open reading frames (ORFs) were amplified in multiple independent reactions using ExTaq DNA polymerase and sequence verified. The consensus nucleotide sequence data are available in the GenBank database under the accession numbers: AEK80438 (LhGαi), AEK80436 (LhGαs), and AEK80437 (LhGαq1).

**Table 1:** Oligonucleotide primers used.

Primer	Sequence (5'–3')		Primer	Sequence (5'–3')	
Ga deg 1 F	ACNATNGTNAARCARATG (TIVKQM)	Degenerate PCR	LhGao 679 F	CGACGTGATACAGAGGATG	Transcriptional Expression Profiling
Ga deg 2 F	GAYGTNGGNGGNCARYG (DVGGQR)		LhGao 1,211 R	TTGTCAATGGCGACTTCTT	
Ga deg 3 F	AARTGGATHCAYTGYTT (KWIHCF)		LhGai 499 F	AACTACGTTCCAACTCAGC	
Ga deg 1a R	RTCTTYTTRTTNAGRAA (FLNKKD)		LhGai 1,026 R	ATCAGTGACAGCATCGAAG	
Ga deg 1b R	RTCYTGYTTRTTNAGRAA (FLNKQD)		LhGas 331 F	GTCCGCGTCGACTATATAC	
Ga deg 2 R	TCNGTNACNGCRTCRAANAC (VFDAVTD)		LhGas 862 R	CCTTGATCTTCTCTGCCAG	
LhGaq 468 F1	GGAAATCGATAGAGTGGCAG				
LhGai sp F2	CAAGTGGTTTGTCGAGACTTCC	5' & 3' RACE	LhGaq 474 F2	GGCGAGAATAGAGAGTCCAG	
LhGai sp R1	CATCTTCTGCAAGTACTAGGTCGT		LhGaq 1,036 R1	AAGGTTGTACTCCTTGAGATTT	
LhGai sp R2	TGTTAGTGTCAGTAGCGCAGGT		LhGaq 1,035 R2	CTAGATTGAATTCTTTGAGTGCA	
LhGai sp F2b	GGTTCCAATACGTATGAAGAAGCAG		LhGa12/13 307 F	TTGAGCCGGAATTGATCAA	
LhGaq2 F1	TCCTTGTCGCGCTCAGTGAATACG		LhGa12/13 834 R	CCACGAGAACTTGATCGAA	
LhGaq2 F2	TCGAATCGGAAAATGAGAACCGAATGGA				
LhGaq2 R1	GGACGAGTGCTGGAACCAGGGGTA		LH Gas no stop R	TAGCAACTCATATTGGCG	Cellular Localization
LhGaq2 R2	TCCATTCGGTTCTCATTTTCCGATTCGA		LhGaq no stop R	AACAAGGTTGTACTCCTTGAGA	
LhGas F1	CCGCCATCATATTCGTGACCGCCT		LH Gi no stop R	GAATAGGCCACAATTTTTTAAGTTT	
LhGas F2	AAGACCCCACGCAGAACCGTCTCA				
LhGas R1	TGAGACGGTTCTGCGTGGGGTCTT				
LhGas R2	AGGCGGTCACGAATATGATGGCGG				
LhGas + stop R	TTATAGCAACTCATATTGGCG	Full Length Clones			
LH Gas start F	AAATCGTCATGGGGTGC				
LhGaq start F	AGATGGCGTGCTGTTTG				
LhGaq end R	TTAAACAAGGTTGTACTCCTTGAGA				
LhGi start F	TAATGGGTTGCGCGATCAG				
LhGi end R	TTAGAATAGGCCACAATTTTTTAAGTTT				
LhGao start F	ATGGGCTGTGCAATGTCTG				
LhGao stop R	TTAGTAAAGTCCACAACC				
LhGa12/13 start	ATGGCGAGTGATATATTTTG				
LhGa12/13 stop	TCATTGCAACATGAGGGAT				

To identify additional Gα subunits and potential variants of the LhGα subunits identified above, *L. hesperus* transcriptomes [43,46], which became available after the initiation of the LhGα cloning project, were searched using BLASTx (*E* value ≤ 10^−10^) with queries consisting of the consensus LhGα sequences and other insect Gα subunits. Sequence hits were then re-evaluated against the NCBI nr (non-redundant) database and duplicates removed. This search identified two additional Gα subunits (LhGαo and LhGα12) and three potential LhGαq variants. Primers were designed to the putative start and stop codons of LhGαo and LhGα12 and to unique portions of the LhGαq variants (Table 1). The respective sequences were amplified from multiple independent reactions using Sapphire Amp Fast PCR Master Mix (Takara Bio Inc./Clontech), subcloned where possible into a pCR2.1 TOPO TA cloning vector (Life Technologies) and sequence verified. The nucleotide sequence data are available in the GenBank database under the accession numbers: KM610199-KM610202 (LhGαq2- LhGαq5), KM610203 (LhGα12), and KM610204 (LhGαo).

### 2.3. Bioinformatic Analyses

LhGα sequences were evaluated against the NCBI nr database by BLASTx (*E* value ≤ 10^−5^). Putative myristoylation sites were predicted using NMT-MYR Predictor (http://mendel.imp.ac.at/myristate/SUPLpredictor.htm) and palmitoylation sites with CSS-PALM (http://csspalm.biocuckoo.org/index.php) [64]. To determine potential phylogenetic relationships, multiple sequence alignments of the putative LhGα subunits and other insect Gα subunits (nine per subfamily) were constructed using default settings in MUSCLE [65,66]. Phylogenetic inferences were made using the maximum likelihood, minimum evolution, NJ, and UPGMA modules implemented in MEGA6.06 [67] with bootstrap analysis conducted of 1000 replicates. Data shown are for the maximum likelihood method based on the JTT matrix-based model [68]. Initial tree(s) for the heuristic search were obtained by applying the NJ method to a matrix of pairwise distances estimated using a JTT model. The analysis involved 53 amino acid sequences. All positions containing gaps and missing data were eliminated. There were a total of 350 positions in the final dataset.

### 2.4. Transcriptional Profiling of L. hesperus Gα Subunits

The expression profiles of the respective LhGα transcripts were examined across *L. hesperus* development and within sex-specific adult body tissues. Developmental profiling consisted of eggs, pooled samples from each of the five nymphal instars, and mixed sex adults comprising equal numbers of males and females at 1, 10, and 20 days post-adult emergence. Adult tissue profiling was performed using cDNAs generated from pooled, sex specific virgin 7-day-old adult bodies, heads, midgut/hindgut, Malpighian tubules, antennae, probosci, and legs as well as pooled tissue sets of female ovaries and seminal depositories, and male medial/lateral accessory glands and testes. Samples were homogenized in TRI Reagent Solution (Ambion/Life Technologies) using a TissueLyser (Qiagen, Valencia, CA, USA) with total RNA extracted based on recommendations from the manufacturer. First-strand cDNAs were generated using a Superscript III first-strand cDNA synthesis kit (Life Technologies) with custom-made random pentadecamers (IDT, San Diego, CA, USA) and 500 ng of DNase I-treated total RNAs. End-point PCR amplification was done using Sapphire Amp Fast PCR Master Mix with 0.4 μL (10 ng) cDNA template, sequence-specific primers (Table 1) designed to amplify ~500–600 bp fragments of the LhGα transcripts, and thermocycler conditions described in Figure 1. Both developmental and adult tissue expression profiles were replicated at least three times using cDNA templates prepared from different biological replicates. Differing combinations of primer sets (see Table 1) designed from transcriptomic data were used to profile the LhGαq1–4 variants: LhGαq1 (LhGaq 468 F1/LhGaq 1036 R1), LhGαq2 (LhGaq 468 F1/LhGaq 1035 R2), LhGαq3 (LhGaq 474 F2/LhGaq 1036 R1), and LhGαq4 (LhGaq 474 F2/LhGaq 1035 R2). PCR products were electrophoresed on 1.5% agarose gels and representative amplimers of the expected sizes were sub-cloned and sequence verified.

### 2.5. Immunocytochemical Localization of L. hesperus Gα in Cultured Insect Cells

To examine the intracellular localization of select LhGα subunits, the respective coding sequences lacking endogenous stop codons were amplified from plasmid DNAs using KOD HotStart DNA polymerase (Toyobo/Novagen, EMD Biosciences, San Diego, CA, USA) and sub-cloned into a pIB/V5-His TOPO TA expression vector (Life Technologies) upstream of the plasmid-derived epitope tag such that the translated LhGα subunits contain a carboxyl terminal 6×-His tag. All resulting expression plasmids were sequence verified. Adherent *Trichoplusia ni* (Tni) cells (Orbigen Inc., San Diego, CA, USA) attached to 35-mm #1.5 glass bottom dishes (*In Vitro* Scientific, Sunnyvale, CA, USA) were transfected with 2 μg plasmid DNA using Insect Gene Juice transfection reagent (Novagen) for 5 h. Transfected cells were maintained in serum-free media for 48 h at 28 °C and then fixed for 15 min at 4 °C with 3.5% formalin/IPL-41. The cells were blocked and permeabilized for 1 h at 25 °C in PBS/10% fetal bovine serum/0.1% Triton X-100. The cells were then incubated for 2 h at 25 °C with 1:50 rabbit polyclonal anti-His antibody (Santa Cruz Biotechnology Inc., Santa Cruz, CA, USA; #SC-804), which recognizes the plasmid-derived His epitope tag. After washing, the cells were incubated with 1:100 goat anti-rabbit IgG-TRITC (Southern Biotechnology; Birmingham, AL, USA; #4030-03) for 2 h at 25 °C. Fluorescent imaging was performed on an Olympus FSX-100 fluorescence microscope with FSX-BSW imaging software (Olympus, Center Valley, PA, USA). Images were processed for publication with Adobe Photoshop CS6 (Adobe Systems, San Jose, CA, USA).

## 3. Results and Discussion

### 3.1. Identification of L. hesperus Gα Sequences

To identify Gα proteins expressed in *L. hesperus* (LhGα), we initially utilized a homology-based approach with degenerate primers designed to conserved regions of Gα proteins and both PCR and nested PCR conditions. Sequence analysis indicated amplimers of the expected sizes were partial fragments of proteins homologous with Gαs, Gαi, and Gαq proteins. Further extension of the partial sequences using conventional RACE PCR methods identified putative start and stop codons. Primers designed to those regions facilitated amplification of the respective open reading frames (ORFs). Based on sequence similarities with known Gα subunits (Table 2), we designated the cloned sequences as LhGαi, LhGαs, and LhGαq. The 1230 nt LhGαi transcript contains a 1068 nt ORF encoding a 355 amino acid residue protein, whereas the 1538 nt LhGαq transcript encompasses a 1062 nt ORF encoding a protein containing 353 amino acids. The 1350 nt LhGαs transcript has a 1137 nt ORF encoding a 378 amino acid protein. The predicted molecular masses of the three Gα proteins (LhGαi = 40.6 kDa, LhGαs = 44.2 kDa, and LhGαq = 41.5 kDa) are comparable with previous reports [3].

Because the degenerate primers used in the homology-based PCR approach have the potential to bias toward particular classes of Gα proteins [62,63], we sought to use recently assembled *L. hesperus* transcriptomes [43,46] to more comprehensively evaluate LhGα expression. The respective databases were queried with the LhGαi, LhGαs, and LhGαq sequences as well as Gα subunits from other insects. All three LhGα transcripts are present in the databases with minimal (>99% nt identity) sequence variation. In addition, complete transcripts for Gαo and Gα12 subunits were identified. The putative LhGαo ORF encodes a 355 amino acid protein with highest sequence similarity to a Gαo subunit cloned from a migratory locust (*Locusta migratoria*) head cDNA library [54]. While the putative LhGα12 encodes a 368 amino acid protein that has significant sequence identity with genomic sequences annotated simply as Gα subunit-like proteins (Table 2), it is 63% identical (*E* value = 3*e*^−154^) with the *D. melanogaster* Gα12 homolog, *concertina* [50]. To confirm correct assembly of the transcriptomic data, the complete coding regions for both LhGαo and LhGα12 were amplified from *L. hesperus* cDNAs in multiple independent reactions and sequenced. As before, the cloned sequences exhibited >99% nt sequence identity with the transcriptomic sequences.

**Table 2:** Top five BLASTx hits for LhGα sequences.

Query	Description	Accession	E Value	% identity	% positives
LhGαs	Guanine nucleotide-binding protein G(s) subunit alpha [*Zootermopsis nevadensis*]	KDR14965.1	0.00E + 00	340/379 (90%)	359/379 (94%)
	PREDICTED: guanine nucleotide-binding protein G(s) subunit alpha [*Diaphorina citri*]	XP_008468199.1	0.00E + 00	338/380 (89%)	360/380 (94%)
	PREDICTED: guanine nucleotide-binding protein G(s) subunit alpha [*Acyrthosiphon pisum*]	XP_001944148.1	0.00E + 00	335/380 (88%)	362/380 (95%)
	guanine nucleotide binding protein, alpha stimulating activity polypeptide [*Daphnia pulex*]	EFX88427.1	0.00E + 00	330/379 (87%)	359/379 (94%)
	guanine nucleotide-binding protein G, putative [*Pediculus humanus corporis*]	XP_002431834.1	0.00E + 00	331/380 (87%)	355/380 (93%)
LhGαi	Guanine nucleotide-binding protein G(i) subunit alpha [*Zootermopsis nevadensis*]	KDR22153.1	0.00E + 00	321/355 (90%)	339/355 (95%)
	PREDICTED: guanine nucleotide-binding protein G(i) subunit alpha-like [*Megachile rotundata*]	XP_003707938.1	0.00E + 00	314/355 (88%)	333/355 (93%)
	PREDICTED: G protein alpha i subunit [*Tribolium castaneum*]	XP_008200240.1	0.00E + 00	313/355 (88%)	331/355 (93%)
	PREDICTED: guanine nucleotide-binding protein G(i) subunit alpha-like [*Apis mellifera*]	XP_395172.2	0.00E + 00	311/355 (88%)	331/355 (93%)
	PREDICTED: guanine nucleotide-binding protein G(i) subunit alpha-like [*Bombus terrestris*]	XP_003393073.1	0.00E + 00	310/355 (87%)	330/355 (92%)
LhGαq1	GTP-binding protein alpha subunit, gna [*Anopheles sinensis*]	KFB50356.1	0.00E + 00	336/353 (95%)	343/353 (97%)
	AGAP005079-PI [*Anopheles gambiae* str. PEST]	XP_313956.1	0.00E + 00	336/353 (95%)	343/353 (97%)
	PREDICTED: guanine nucleotide-binding protein G(q) subunit alpha isoform X1 [*Acyrthosiphon pisum*]	XP_001948628.2	0.00E + 00	333/353 (94%)	346/353 (98%)
	GTP-binding protein alpha subunit, gna [*Aedes aegypti*]	XP_001660884.1	0.00E + 00	335/353 (95%)	343/353 (97%)
	PREDICTED: guanine nucleotide-binding protein G(q) subunit alpha-like isoform 1 [*Megachile rotundata*]	XP_003702524.1	0.00E + 00	335/353 (95%)	344/353 (97%)
LhGαq2	PREDICTED: G protein alpha q subunit isoform X2 [*Acyrthosiphon pisum*]	XP_008178833.1	0.00E + 00	322/353 (91%)	337/353 (95%)
	AGAP005079-PB [Anopheles gambiae str. PEST]	XP_001688493.1	0.00E + 00	325/353 (92%)	334/353 (94%)
	PREDICTED: guanine nucleotide-binding protein G(q) subunit alpha-like isoform 5 [*Megachile rotundata*]	XP_003702528.1	0.00E + 00	318/353 (90%)	338/353 (95%)
	PREDICTED: guanine nucleotide-binding protein G(q) subunit alpha-like isoform X5 [*Apis mellifera*]	XP_006562642.1	0.00E + 00	319/353 (90%)	334/353 (94%)
	PREDICTED: guanine nucleotide-binding protein G(q) subunit alpha-like isoform X13 [*Apis dorsata*]	XP_006615865.1	0.00E + 00	318/353 (90%)	333/353 (94%)
LhGαq3	PREDICTED: guanine nucleotide-binding protein G(q) subunit alpha-like isoform 2 [*Megachile rotundata*]	XP_003702525.1	0.00E + 00	334/353 (95%)	343/353 (97%)
	PREDICTED: guanine nucleotide-binding protein G(q) subunit alpha isoform X3 [*Acyrthosiphon pisum*]	XP_008178834.1	0.00E + 00	331/353 (94%)	345/353 (97%)
	AGAP005079-PF [Anopheles gambiae str. PEST]	XP_001688489.1	0.00E + 00	333/353 (94%)	343/353 (97%)
	PREDICTED: guanine nucleotide-binding protein G(q) subunit alpha-like isoform X8 [*Apis mellifera*]	XP_623211.2	0.00E + 00	328/353 (93%)	341/353 (96%)
	PREDICTED: guanine nucleotide-binding protein G(q) subunit alpha-like isoform 1 [*Megachile rotundata*]	XP_003702524.1	0.00E + 00	325/353 (92%)	338/353 (95%)
LhGαq4	PREDICTED: guanine nucleotide-binding protein G(q) subunit alpha-like isoform 3 [*Megachile rotundata*]	XP_003702526.1	0.00E + 00	324/353 (92%)	334/353 (94%)
	G protein alpha q isoform 2 [*Bombyx mori*]	NP_001128385.1	0.00E + 00	322/353 (91%)	335/353 (94%)
	PREDICTED: G protein alpha q subunit isoform X4 [*Acyrthosiphon pisum*]	XP_008178835.1	0.00E + 00	320/353 (91%)	336/353 (95%)
	GTP-binding protein alpha subunit, gna [*Aedes aegypti*]	XP_001660885.1	0.00E + 00	322/353 (91%)	334/353 (94%)
	AGAP005079-PD [Anopheles gambiae str. PEST]	XP_001688487.1	0.00E + 00	322/353 (91%)	334/353 (94%)
LhGαq5	PREDICTED: G protein alpha q subunit-like [*Diaphorina citri*]	XP_008479779.1	4.00E − 102	145/157 (92%)	151/157 (96%)
	PREDICTED: G protein alpha q subunit isoform X4 [*Acyrthosiphon pisum*]	XP_008178835.1	2.00E − 99	152/193 (79%)	155/193 (80%)
	PREDICTED: guanine nucleotide-binding protein G(q) subunit alpha-like isoform 3 [*Megachile rotundata*]	XP_003702526.1	2.00E − 99	153/193 (79%)	155/193 (80%)
	PREDICTED: guanine nucleotide-binding protein G(q) subunit alpha isoform X3 [*Tribolium castaneum*]	XP_008195246.1	1.00E − 98	152/193 (79%)	155/193 (80%)
	PREDICTED: guanine nucleotide-binding protein G(q) subunit alpha isoform X5 [*Acyrthosiphon pisum*]	XP_008178836.1	2.00E − 98	152/193 (79%)	155/193 (80%)
LhGα12	Guanine nucleotide-binding protein subunit alpha-like protein [*Harpegnathos saltator*]	EFN86700.1	0.00E + 00	287/367 (78%)	327/367 (89%)
	PREDICTED: guanine nucleotide-binding protein subunit alpha homolog [*Apis mellifera*]	XP_394382.2	0.00E + 00	286/367 (78%)	326/367 (88%)
	PREDICTED: guanine nucleotide-binding protein subunit alpha homolog [*Nasonia vitripennis*]	XP_001600076.1	0.00E + 00	282/363 (78%)	324/363 (89%)
	Guanine nucleotide-binding protein subunit alpha-like protein [*Acromyrmex echinatior*]	EGI64184.1	0.00E + 00	288/368 (78%)	328/368 (89%)
	PREDICTED: guanine nucleotide-binding protein subunit alpha homolog [*Bombus terrestris*]	XP_003402866.1	0.00E + 00	283/367 (77%)	325/367 (88%)
LhGαo	Guanine nucleotide-binding protein G(o) subunit alpha [*Locusta migratoria*]	P38404.1	0.00E + 00	346/354 (98%)	349/354 (98%)
	Guanine nucleotide-binding protein G(o) subunit alpha [*Zootermopsis nevadensis*]	KDR16702.1	0.00E + 00	345/354 (97%)	348/354 (98%)
	Guanine nucleotide-binding protein G(o) subunit alpha [*Camponotus floridanus*]	EFN66163.1	0.00E + 00	344/354 (97%)	348/354 (98%)
	PREDICTED: guanine nucleotide-binding protein G(o) subunit alpha-like isoform 1 [*Megachile rotundata*]	XP_003701784.1	0.00E + 00	342/354 (97%)	347/354 (98%)
	PREDICTED: guanine nucleotide-binding protein G(o) subunit alpha [*Microplitis demolitor*]	XP_008545405.1	0.00E + 00	339/354 (96%)	346/354 (97%)

**Figure 2:**
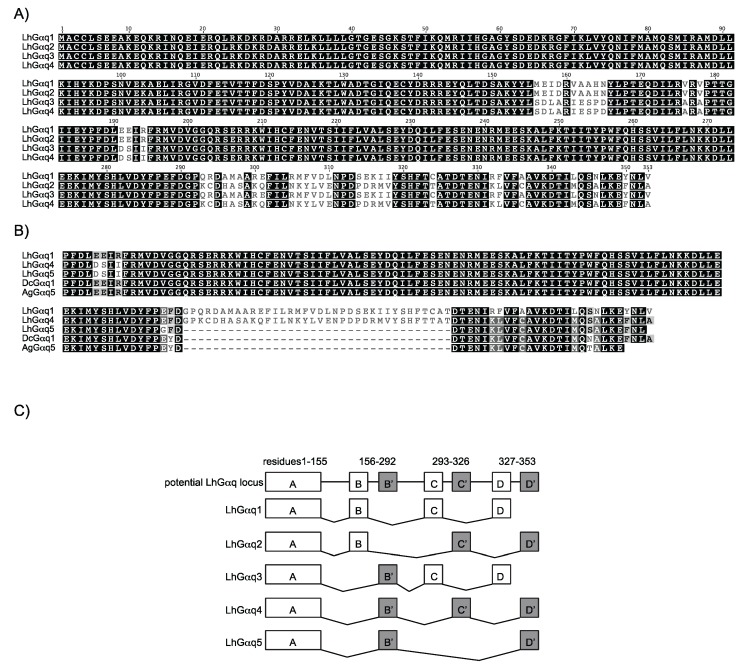
Alternative splice variants of LhGαq. (**A**) MUSCLE-based sequence alignment of LhGαq1-4; (**B**) MUSCLE-based sequence alignment of LhGαq5 with LhGαq1 and Gαq variants from *A. gambiae* (AAW50316; AgGαq5) and *Diaphorina citri* (XP_008479779; DcGαq1). The alignment corresponds to LhGαq1 amino acid residues 187–353. Shading denotes amino acid sequence similarity and is scaled as: 100% similarity (black), 99%–80% (dark grey), 79%–60% (light grey), and less than 59% (white); (**C**) Proposed alternative splice scheme utilized to generate the cloned LhGαq variants. Similar to *A. gambiae* and *D. melanogaster*, the LhGαq locus appears to have three homologous exons (B/B', C/C', and D/D') that are alternatively spliced to generate the five LhGαq variants. Putative exon-intron boundaries are based on observed sequence variations with coding sequences shown as boxes.

Multiple sequence variants have been reported for Gα subunits [20,48,51,52,69,70] with variants/isoforms also predicted in many insect genomes. Furthermore, high throughput sequencing methods, such as those used to construct the *L. hesperus* transcriptome databases, offer the possibility of identifying low representation and/or unique transcripts [71,72,73]. Consistent with previous findings, our transcriptome database search identified three additional LhGαq variants, which we have designated LhGαq2-4. Sequence identity among the four subunits varies from 89%–96% with all four variants identical through Leu155, at which point identity is maintained between LhGαq1/2 and LhGαq3/4 up to Pro292, with identical residues then shared between LhGαq1/3 and LhGαq2/4 throughout the rest of the protein (Figure 2A). This variation is consistent with the alternative exon splicing described in *A. gambiae* [20] and *D. melanogaster* [70] and is present in a number of species from disparate orders, suggesting that the putative splice sites have been evolutionarily conserved. While characterizing the respective LhGαq variants, we cloned a partial sequence corresponding to a fifth variant (LhGαq5) that is not represented in either of the transcriptomic databases and which lacks residues 291–326 (Figure 2B)*.* While this variant is also present in *A. gambiae* (AAW50316) and *Diaphorina citri* (XP_008479779) (Figure 2B), we were unable to identify it from other insects, which suggests that the splice site is either not conserved or that it is a cryptic site [73]. The Gαq locus in *A. gambiae* spans 11 exons, three of which (identified as D/D*, G/G*, and H/H*) are homologous and undergo alternative splicing [20]. While the genomic structure of the *L. hesperus* Gαq locus has not been determined, we can surmise based on the transcript sequences that similar alternative splicing likely generates the five variants (Figure 2C).

In their characterization of Gα in *A. gambiae*, Rützler *et al.* [20] identified a sixth Gαq variant (AAW50317) characterized by inclusion of a 43 amino acid insertion that corresponds to two of the exons alternatively spliced in the other variants. Although this variant is a predicted product in a number of insect genomes, it was considered to be a premature transcript as inclusion of the second exon could potentially disrupt the catalytic pocket of the GTP hydrolysis domain. We were unable to detect this variant during characterization of the other LhGαq subunits nor was it represented in the *L. hesperus* transcriptomes [43,46]. No other LhGα sequence variants were identified. The respective transcriptomes, however, may underrepresent the number of Gα transcripts actively expressed in *L. hesperus* due to the exclusion of temporally or spatially restricted transcripts.

### 3.2. Bioinformatic Analysis of LhGα Subunits

**Figure 3:**
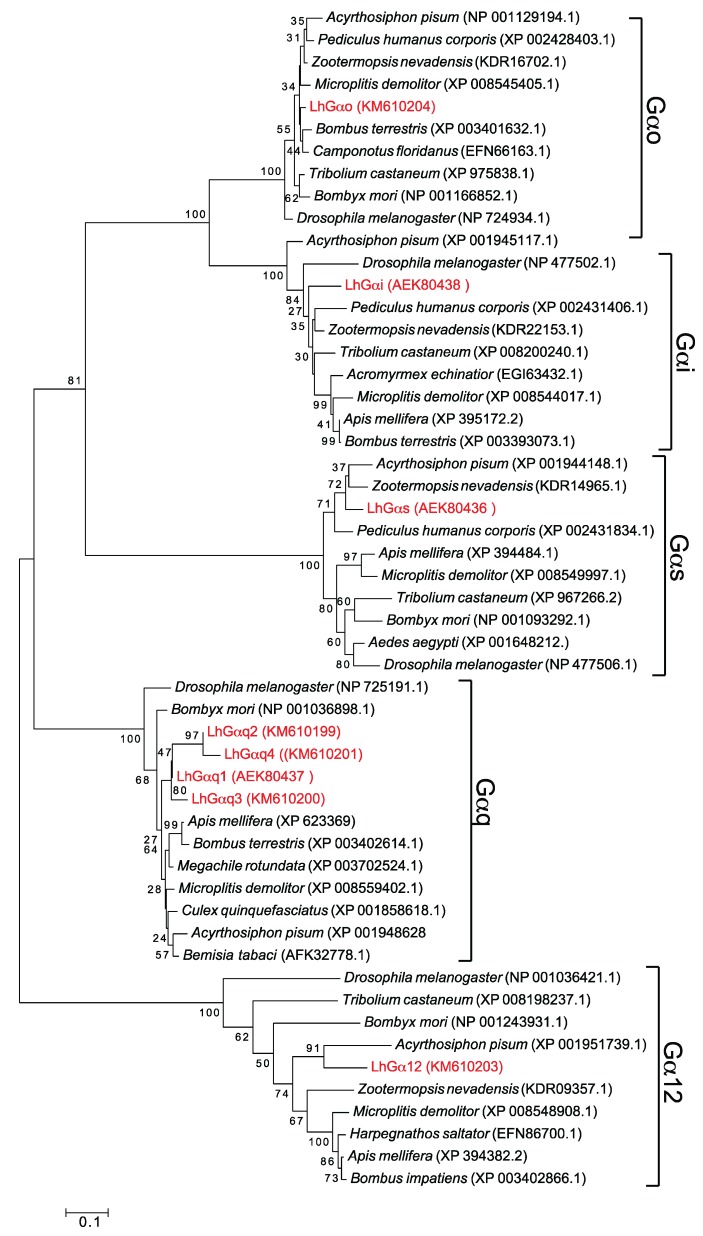
Phylogenetic analysis of Gα subunits from *L. hesperus* and other insects. Phylogenetic relationships were inferred using the maximum likelihood method based on the JTT matrix-based model [68]. The tree with the highest log likelihood is shown. *L. hesperus* Gα sequences are shown in red.

Sequence identity across the respective full-length LhGα sequences ranges from 33%–96% (Table 3) with the highest values associated, as expected, with the LhGαq variants. Sequence identity across the subfamilies varied from 35%–67%, with highest identity shared between LhGαo and LhGαi, which is consistent with previous studies showing that Gαo and Gαi are phylogenetically related [1,3]. BLASTx analyses using the nr database revealed the highest similarities (Table 2) were predominantly with non-hemipteran sequences, indicating the highly conserved evolutionary nature and functional importance of Gα subunits and the current lack of molecular resources for hemipteran pests. Sequence alignment and phylogenetic analyses of the putative LhGα proteins with those from other arthropods (Figure 3) support our initial classifications and are consistent with previously reported evolutionary relationships [20,51,57]. Based on their conserved structural similarities, the respective Gα classes were divided into five central clades with strong bootstrap support for a shared branch point between the Gαo and Gαi clades.

**Table 3:** Percent identity matrix heat map for LhGα proteins.

	LhGαq1	LhGαq2	LhGαq3	LhGαq4	LhGαq5	LhGαs	LhGαi	LhGαo	LhGα12
LhGαq1	100	93	96	89	72	42	49	48	44
LhGαq2	93	100	89	96	75	42	47	46	44
LhGαq3	96	89	100	93	78	42	50	48	45
LhGαq4	89	96	93	100	81	42	48	46	44
LhGαq5	72	75	78	81	100	39	47	47	43
LhGαs	42	42	42	42	39	100	41	42	35
LhGαi	49	47	50	48	47	41	100	67	38
LhGαo	48	46	48	46	47	42	67	100	39
LhGα12	44	44	45	44	43	35	38	39	100

**Figure 4:**
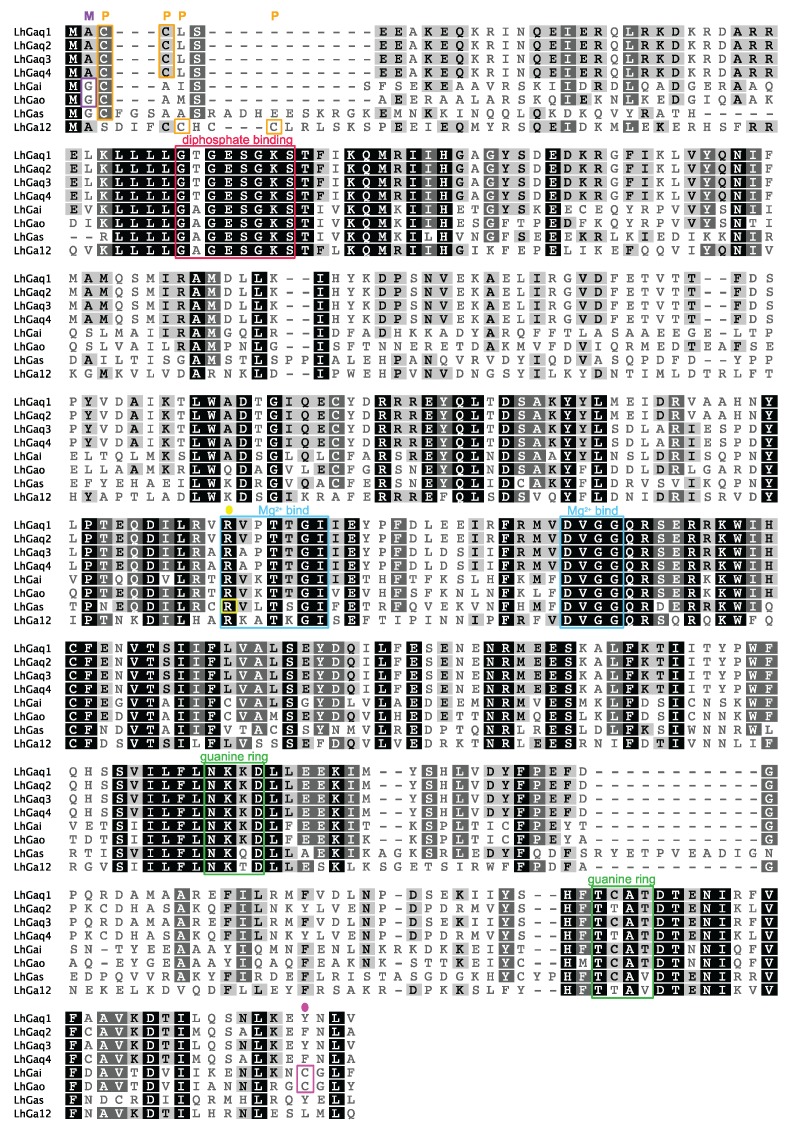
Multiple sequence alignment of *L. hesperus* Gα sequences. The respective full length *L. hesperus* Gα sequences were aligned in MUSCLE using default settings. Percent similarity shading is as in Figure 2. Conserved Gα subunit features/motifs are boxed: predicted myristoylation sites (purple M), predicted palmitoylation sites (orange P), diphosphate binding (red), Mg^2+^ binding (blue), guanine ring binding (green), putative cholera toxin-mediated ADP-ribosylation site (Arg186) in LhGαs (yellow circle), putative pertussis toxin-mediated ADP-ribosylation sites (Cys352/Cys351) in LhGαi and LhGαo (pink circle).

The conserved guanine nucleotide binding/hydrolysis motifs characteristic of Gα subunits are present in the predicted LhGα proteins (Figure 4) including sequences critical for diphosphate binding (GXGESGKS), Mg^2+^ binding (RXXTXGI and DXXG), and guanine ring-binding (NKXD and TCAT) [3]. Deviations from the canonical sequences, however, are present in the TCAT motif in LhGαs (TCAV), LhGα12 (TTAV), and LhGαq2/4 (TTAT). These deviations are not specific to the *L. hesperus* sequences as all of the Gαs and Gα12 sequences used in the phylogenetic analysis had the same sequence changes and numerous Gαq sequences (e.g., NP_001128385, *B. mori*; ACJ06653, *Spodoptera frugiperda*; CAB76453, *Calliphora vicina*; XP_005180085, *Musca domestica*; XP_004526037, *Ceratitis capitata*) have a TTAT motif. Mutations to the TCAT motif in mammalian Gα subunits mimic an activated receptor by enhancing GDP release [74,75]. Thus, activation of insect Gα subunits with the modified TCAT motif may proceed more readily, which could account for the observed heterogeneity in receptor-G protein interactions and promiscuous activation of multiple Gα subunits by some receptors [1].

Further analysis of the LhGα sequences indicated the presence of conserved modification sites for fatty acids and toxin-driven ADP-ribosylation (Figure 4). Palmitoylation of Gα amino terminal Cys residues and/or myristoylation of amino terminal Gly residues in Gαi/o subunits can influence cellular localization/membrane targeting, interactions with downstream effector proteins, and secondary structure [2,3,76]. ADP-ribosylation of a carboxyl terminal Cys by pertussis toxin uncouples Gαi/o subunits whereas similar modification of an internal Arg in Gαs subunits by cholera toxin abolishes GTP hydrolysis activity and leads to constitutive Gαs activation [1,3].

The last five residues of the Gα carboxyl terminus are critical for receptor interactions, with minor modifications of this region altering receptor specificity and ADP-ribosylation uncoupling Gαi/o subunits from the respective receptor [2]. The identical carboxyl terminal ends shared by LhGαq1/3 and LhGαq2/4 raises questions regarding potentially overlapping functional roles. One possibility is that the respective subunits exhibit different expression profiles (see below), which would limit functional redundancy. A second possibility is that the sequence variations that differentiate the respective LhGαq subunits also function to stabilize receptor interactions. Thus, despite identical carboxyl terminal ends the LhGαq subunits interact with the receptors differently. Consequently, despite the critical role the carboxyl terminus plays, functional specificity is driven by the summation of receptor contact points.

### 3.3. End Point PCR-Based Transcriptional Expression Profiling

**Figure 5:**
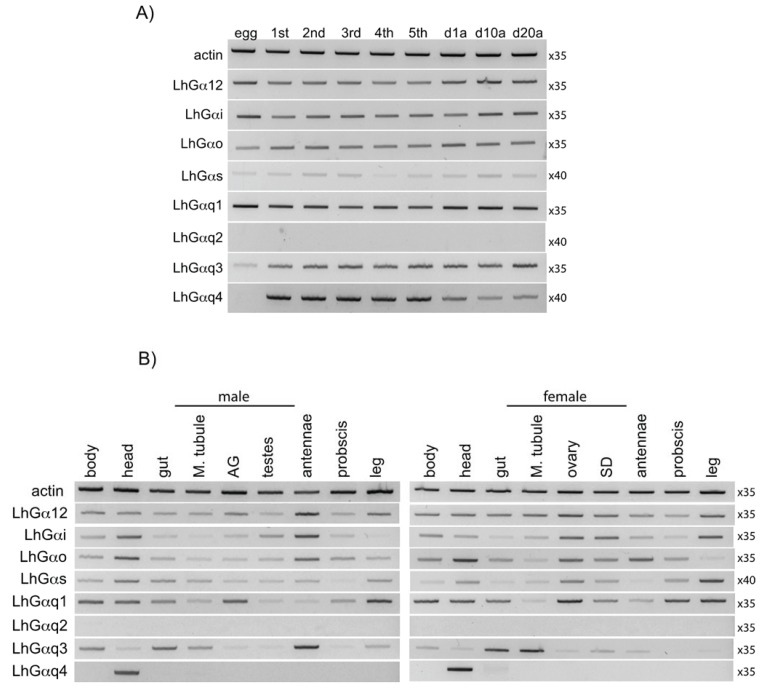
End point PCR-based expression profile of *L. hesperus* Gα transcripts. (**A**) Expression profile of LhGα sequences in eggs, nymphal development (1st–5th instars), 1-day-old mixed sex adults (d1a), 10-day-old mixed sex adults (d10a), and 20-day-old mixed sex adults (d20a); (**B**) Expression profile of LhGα sequences in sex-specific adult tissues. Abbreviations are: M. tubule, Malpighian tubule; AG, accessory glands (lateral and medial); SD, seminal depository. In both (**A**) and (**B**), amplimers correspond to ~500-600 bp fragments of each transcript with products analyzed on 1.5% agarose gels stained with SYBR Safe. Negative images of the gels are shown for enhanced clarity of low expression transcripts. Numbers to the right of each gel image indicate the number of amplification cycles.

The tissue and/or developmental specificity of transcript expression can provide insights into gene functionality. To begin to assess the potential functional role of the LhGα subunits, we examined their transcriptional expression as ~500–600 bp fragments across *L. hesperus* development, from eggs through 5th instars and in 1-day-old, 10-day-old, and 20-day-old adults (Figure 5A). While most LhGα subunits were ubiquitously expressed in all stages examined, the expression of LhGαq2 and LhGαq4 was more restrictive. The LhGαq4 product was absent in eggs but was detected throughout nymphal development and in adults (Figure 5A). Even though LhGαq2 and LhGαq4 share identical carboxyl terminal ends (see above), no amplimers were detected for LhGαq2, suggesting little functional redundancy with respect to receptor specificity between the two variants. Despite overlap with the LhGαq4 primer set (as demonstrated by the serendipitous cloning of LhGαq5 while verifying the LhGαq4 sequence), no LhGαq5 amplimers, which would migrate as a lower molecular weight product (*i.e.*, 476 bp *vs*. 584 bp for LhGαq4) were detected, suggesting low transcript levels for this variant. 

We also examined the expression profile of the LhGα subunit fragments in sex-specific adult tissues (Figure 5B). A majority of the LhGα transcripts were amplified from all of the tissue sets from both sexes, albeit to varying degrees. Similarly wide tissue distribution profiles for Gα subunits have been reported in *B. mori* [19], *A. gambiae* [20], *D. melanogaster* [21], *B. tabaci* [57], and *L. oryzophilus* [55] and likely reflect the critical role of G proteins in mediating the diverse signal transduction cascades that drive cellular processes. LhGαq4 was the lone LhGα transcript to exhibit tissue specific expression with amplification limited to head-derived cDNAs (Figure 5B). LhGαq4 shares significant sequence identity with *D. melanogaster* Gαq1 (*i.e.*, Gq-RD), the Gα subunit involved in phototransduction [77,78], and the presumptive *A. gambiae* ortholog, Agq1 [20]. All three are derived from analogous alternative splice sites and are specifically expressed in adult heads and pre-adult stages with no detectable embryonic expression [20,21,77]. These similarities suggest that functionality may also be conserved, with LhGαq4 likewise mediating phototransduction. This, however, remains to be experimentally verified. No amplimers corresponding to LhGαq2 were detected in any of the tissues examined, which is consistent with the developmental expression profile (Figure 5A).

With the exception of LhGαq2 and LhGαq4, all of the LhGα subunits were amplified to varying degrees from chemosensory tissues (antenna, proboscis, and leg) indicating the absence of a chemosensory specific subunit (Figure 5B). While variation in amplification across the chemosensory tissues was observed for LhGαo (highest in antennae) and LhGαq1 (highest in leg), more accurate determinations (e.g., quantitative real time-PCR) of transcript abundance are required to draw definitive conclusions regarding expression. Gαs has been reported to be more highly expressed in antennae than other Gα subunits in *A. gambiae*, *D. melanogaster*, and *B. mori* [19,20,21]. The expression of Gαs in olfactory neurons coupled with abnormal olfactory behavior following disruption of the Gαs signal transduction cascade [25] has led some to postulate that Gαs functions in olfaction. However, elevated levels of Gαo and Gαq transcripts have been reported in antennae and olfactory neurons of a number of insects [17,18,19,54,55]. Furthermore, similar to the Gαs pathway, downstream effectors of Gαq such as Ca^2+^/calmodulin can also activate adenylyl cyclase [79] and RNAi-mediated knockdown of Gαq likewise reduces antennal responses [29]. In contrast, other studies have suggested that Gα proteins have little role in insect olfaction [35]. Given the conflicting conclusions drawn by disparate groups and the critical role of Gα proteins in normal cellular function, it is becoming increasingly clear that simple co-localization of Gα transcripts within chemosensory tissues, while correlational, is not indicative in and of itself of an olfactory function.

### 3.4. Intracellular Localization of Transiently Expressed LhGα Subunits

**Figure 6:**
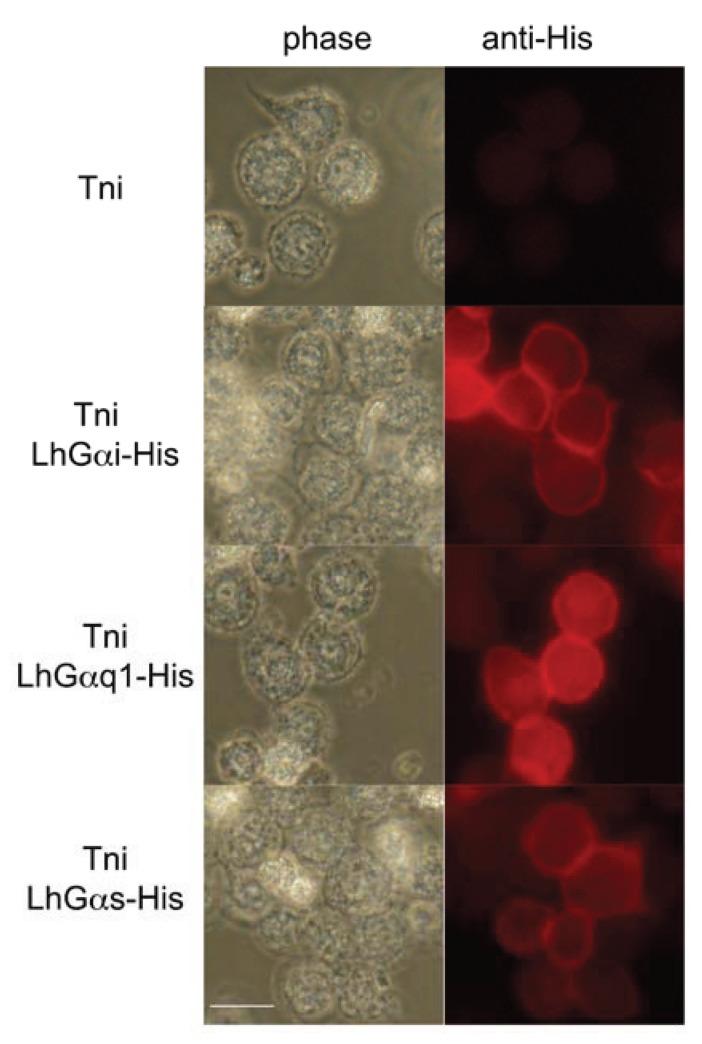
Intracellular localization of transiently expressed *L. hesperus* Gα subunits in cultured insect cells. Fixed *Trichoplusia ni* (Tni) cells transfected with plasmids encoding carboxyl terminal 6×-His tagged LhGα subunits (LhGαi-His, LhGαq1-His, and LhGαs-His) or mock transfected cells (Tni) were probed with a polyclonal mouse anti-His antibody (primary) and a goat anti-mouse IgG-TRITC antibody (secondary). Red fluorescence corresponds to the TRITC signal and denotes localization of the His-tagged Gα subunits. Scale bar = 20 μm.

Post-translational lipid modifications (*i.e.*, myristolation/palmitoylation) facilitate targeting and subsequent anchoring of Gα subunits to the inner surface of the plasma membrane [76,80]. To further characterize and confirm the sequence validity of the cloned LhGα transcripts, we sought to examine the intracellular localization of a subset of the LhGα proteins (LhGαq1, LhGαs, and LhGαi) following transient expression in cultured insect cells. To facilitate detection, expression vectors were constructed in which a 6×-His tag was incorporated in frame with the carboxyl terminal ends of the respective LhGα sequences. Immunofluorescence analyses were performed in cultured *Trichoplusia ni* cells 48 h after transfection using a polyclonal anti-His antibody in conjunction with a TRITC-tagged anti-rabbit antibody. No fluorescence was observed in non-transfected cells (Figure 6). In contrast, plasma membrane-associated fluorescence was clearly observed in cells transfected with the respective LhGα-His constructs (Figure 6). These results are consistent with previous findings [12] and indicate that intracellular trafficking of the cloned LhGα sequences is as expected. In addition to the clear plasma membrane-associated signal, we also observed a diffuse red fluorescent signal throughout the cytosol of cells transfected with the respective LhGα subunits. The current model of G protein trafficking suggests that interactions between Gα subunits and Gβγ subunits are crucial for plasma membrane localization. Consequently, overexpression of one subunit (e.g., Gα subunits) may disrupt the necessary stoichiometry and lead to inefficient localization [76]. Thus, the intracellular signal we observed might be “free” Gα subunits that lack the apparent Gβγ binding partners that facilitate plasma membrane localization. Alternatively, the signal may represent the normal trafficking profile of Gα subunits as both cell membrane and discrete cytosolic localization for Gα subunits have been reported in both native tissue and cell culture [81,82,83].

## 4. Conclusions

As part of our continuing efforts to further elucidate molecular mechanisms driving signal transduction in *L. hesperus*, we identified nine Gα subunits. Expression analyses and sequence similarities strongly suggest that LhGαq4 is orthologous to *D. melanogaster* Gq-RD, which functions in phototransduction. While the presence of multiple LhGα transcripts in chemosensory tissues is consistent with potential roles in olfaction and/or gustation, localization at the tissue level alone does not imply function in chemosensory-based signal transduction. To address that issue, the actual role of each of the LhGα subunits and variants in chemosensory functionality must be established, including demonstration of specific expression of LhGα within olfactory/gustatory receptor neurons and *in vivo* functional studies examining the biological effects of Gα mutations, Gα knockdown, and/or Gα overexpression. 

## References

[1] Offermanns S. (2003). G-proteins as transducers in transmembrane signalling. Prog. Biophys. Mol. Biol..

[2] Cabrera-Vera T.M., Vanhauwe J., Thomas T.O., Medkova M., Preininger A., Mazzoni M.R., Hamm H.E. (2003). Insights into G protein structure, function, and regulation. Endocr. Rev..

[3] Oldham W.M., Hamm H.E. (2006). Structural basis of function in heterotrimeric G proteins. Q. Rev. Biophys..

[4] Kelly P., Casey P.J., Meigs T.E. (2007). Biologic functions of the G12 subfamily of heterotrimeric g proteins: Growth, migration, and metastasis. Biochemistry.

[5] Worzfeld T., Wettschureck N., Offermanns S. (2008). G12/G13-mediated signalling in mammalian physiology and disease. Trends Pharmacol. Sci..

[6] Nakagawa T., Vosshall L.B. (2009). Controversy and consensus: Noncanonical signaling mechanisms in the insect olfactory system. Curr. Opin. Neurobiol..

[7] Stengl M., Funk N.W. (2013). The role of the coreceptor Orco in insect olfactory transduction. J. Comp. Physiol. A.

[8] Wistrand M., Käll L., Sonnhammer E.L.L. (2006). A general model of G protein-coupled receptor sequences and its application to detect remote homologs. Protein Sci..

[9] Benton R., Sachse S., Michnick S.W., Vosshall L.B. (2006). Atypical membrane topology and heteromeric function of *Drosophila* odorant receptors *in vivo*. PLOS Biol..

[10] Lundin C., Käll L., Kreher S.A., Kapp K., Sonnhammer E.L., Carlson J.R., von Heijne G., Nilsson I. (2007). Membrane topology of the *Drosophila* OR83b odorant receptor. FEBS Lett..

[11] Zhang H.-J., Anderson A.R., Trowell S.C., Luo A.-R., Xiang Z.-H., Xia Q.-Y. (2011). Topological and functional characterization of an insect gustatory receptor. PLOS ONE.

[12] Hull J.J., Hoffmann E.J., Perera O.P., Snodgrass G.L. (2012). Identification of the western tarnished plant bug (*Lygus hesperus*) olfactory co-receptor Orco: Expression profile and confirmation of atypical membrane topology. Arch. Insect Biochem. Physiol..

[13] Sato K., Pellegrino M., Nakagawa T., Nakagawa T., Vosshall L.B., Touhara K. (2008). Insect olfactory receptors are heteromeric ligand-gated ion channels. Nature.

[14] Smart R., Kiely A., Beale M., Vargas E., Carraher C., Kralicek A.V., Christie D.L., Chen C., Newcomb R.D., Warr C.G. (2008). Drosophila odorant receptors are novel seven transmembrane domain proteins that can signal independently of heterotrimeric G proteins. Insect Biochem. Mol. Biol..

[15] Sato K., Tanaka K., Touhara K. (2011). Sugar-regulated cation channel formed by an insect gustatory receptor. Proc. Natl. Acad. Sci. USA.

[16] Wicher D., Schäfer R., Bauernfeind R., Stensmyr M.C., Heller R., Heinemann S.H., Hansson B.S. (2008). *Drosophila* odorant receptors are both ligand-gated and cyclic-nucleotide-activated cation channels. Nature.

[17] Talluri S., Bhatt A., Smith D.P. (1995). Identification of a *Drosophila* G protein alpha subunit (dGq alpha-3) expressed in chemosensory cells and central neurons. Proc. Natl. Acad. Sci. USA.

[18] Jacquin-Joly E., Francois M.-C., Burnet M., Lucas P., Bourrat F., Maida R. (2002). Expression pattern in the antennae of a newly isolated lepidopteran Gq protein alpha subunit cDNA. Eur. J. Biochem..

[19] Miura N., Atsumi S., Tabunoki H., Sato R. (2005). Expression and localization of three G protein alpha subunits, Go, Gq, and Gs, in adult antennae of the silkmoth (*Bombyx mori*). J. Comp. Neurol..

[20] Rützler M., Lu T., Zwiebel L.J. (2006). Galpha encoding gene family of the malaria vector mosquito *Anopheles gambiae*: Expression analysis and immunolocalization of AGalphaq and AGalphao in female antennae. J. Comp. Neurol..

[21] Boto T., Gomez-Diaz C., Alcorta E. (2010). Expression analysis of the 3 G-protein subunits, Galpha, Gbeta, and Ggamma, in the olfactory receptor organs of adult *Drosophila melanogaster*. Chem. Senses.

[22] Deng Y., Zhang W., Farhat K., Oberland S., Gisselmann G., Neuhaus E.M. (2011). The stimulatory Gα(s) protein is involved in olfactory signal transduction in *Drosophila*. PLOS ONE.

[23] Ziegelberger G., van den Berg M.J., Kaissling K.E., Klumpp S., Schultz J.E. (1990). Cyclic GMP levels and guanylate cyclase activity in pheromone-sensitive antennae of the silkmoths *Antheraea polyphe**mus* and *Bombyx mori*. J. Neurosci..

[24] Boekhoff I., Seifert E., Göggerle S., Lindemann M., Krüger B.-W., Breer H. (1993). Pheromone-induced second-messenger signaling in insect antennae. Insect Biochem. Mol. Biol..

[25] Gomez-Diaz C., Martin F., Alcorta E. (2004). The cAMP transduction cascade mediates olfactory reception in *Drosophila melanogaster*. Behav. Genet..

[26] Chatterjee A., Roman G., Hardin P.E. (2009). Go contributes to olfactory reception in *Drosophila melanogaster*. BMC Physiol..

[27] Raja J.S.I., Katanayeva N., Katanaev V.L., Galizia C.G. (2014). Role of G o/i subgroup of G proteins in olfactory signaling of *Drosophila melanogaster*. Eur. J. Neurosci..

[28] Kalidas S., Smith D.P. (2002). Novel genomic cDNA hybrids produce effective RNA interference in adult *Drosophila*. Neuron.

[29] Kain P., Chakraborty T.S., Sundaram S., Siddiqi O., Rodrigues V., Hasan G. (2008). Reduced odor responses from antennal neurons of G(q)alpha, phospholipase Cbeta, and rdgA mutants in *Drosoph**ila* support a role for a phospholipid intermediate in insect olfactory transduction. J. Neurosci..

[30] Ouyang Q., Sato H., Murata Y., Nakamura A., Ozaki M., Nakamura T. (2009). Contribution of the inositol 1,4,5-trisphosphate transduction cascade to the detection of “bitter” compounds in blowflies. Comp. Biochem. Physiol. Part A.

[31] Ishimoto H., Takahashi K., Ueda R., Tanimura T. (2005). G-protein gamma subunit 1 is required for sugar reception in *Drosophila*. EMBO J..

[32] Ueno K., Kohatsu S., Clay C., Forte M., Isono K., Kidokoro Y. (2006). Gsalpha is involved in sugar perception in *Drosophila melanogaster*. J. Neurosci..

[33] Kain P., Badsha F., Hussain S.M., Nair A., Hasan G., Rodrigues V. (2010). Mutants in phospholipid signaling attenuate the behavioral response of adult *Drosophila* to trehalose. Chem. Senses.

[34] Bredendiek N., Hütte J., Steingräber A., Hatt H., Gisselmann G., Neuhaus E.M. (2011). Goa is involved in sugar perception in Drosophila. Chem. Senses.

[35] Yao C.A., Carlson J.R. (2010). Role of G-proteins in odor-sensing and CO_2_-sensing neurons in *Dros**ophila*. J. Neurosci..

[36] Devambez I., Agha M.A., Mitri C., Bockaert J., Parmentier M.-L., Marion-Poll F., Grau Y., Soustelle L. (2013). Gαo is required for l-canavanine detection in *Drosophila*. PLoS One.

[37] Scott D.R. (1977). An annotated listing of host plants of *Lygus hesperus* Knight. Entomol. Soc. Am. Bull..

[38] Young O.P. (1986). Host plants of the tarnished plant bug, *Lygus lineolaris* (Heteroptera: Miridae). Ann. Entomol. Soc. Am..

[39] Blackmer J.L., Rodriguez-Saona C., Byers J.A., Shope K.L., Smith J.P. (2004). Behavioral response of *Lygus hesperus* to conspecifics and headspace volatiles of alfalfa in a Y-tube olfactometer. J. Chem. Ecol..

[40] Blackmer J.L., Canas L.A. (2005). Visual cues enhance the response of *Lygus hesperus* (Heteroptera: Miridae) to volatiles from host plants. Environ. Entomol..

[41] Williams L., Blackmer J.L., Rodriguez-Saona C., Zhu S. (2010). Plant volatiles influence electrophysiological and behavioral responses of *Lygus hesperus*. J. Chem. Ecol..

[42] Byers J.A., Fefer D., Levi-Zada A. (2013). Sex pheromone component ratios and mating isolation among three *Lygus* plant bug species of North America. Naturwissenschaften.

[43] Hull J.J., Geib S.M., Fabrick J.A., Brent C.S. (2013). Sequencing and de novo assembly of the western tarnished plant bug (*Lygus hesperus*) transcriptome. PLOS ONE.

[44] Magalhaes L.C., van Kretschmar J.B., Donohue K.V., Roe R.M. (2013). Pyrosequencing of the adult tarnished plant bug, *Lygus lineolaris*, and characterization of messages important in metabolism and development. Entomol. Exp. Appl..

[45] Hull J.J., Perera O.P., Snodgrass G.L. (2014). Cloning and expression profiling of odorant-binding proteins in the tarnished plant bug, *Lygus lineolaris*. Insect Mol. Biol..

[46] Hull J.J., Chaney K., Geib S.M., Fabrick J.A., Brent C.S., Walsh D., Lavine L.C. (2014). Transcriptome-based identification of ABC transporters in the western tarnished plant bug *Lygus hesperus*. PLOS ONE.

[47] Dickens J.C., Callahan F.E., Wergin W.P., Murphy C.A., Vogt R.G. (1998). Intergeneric distribution and immunolocalization of a putative odorant-binding protein in true bugs (Hemiptera, Heteroptera). J. Exp. Biol..

[48] Quan F., Forte M.A. (1990). Two forms of *Drosophila melanogaster* Gs alpha are produced by alternate splicing involving an unusual splice site. Mol. Cell Biol..

[49] Lee Y.J., Dobbs M.B., Verardi M.L., Hyde D.R. (1990). dgq: A *Drosophila* gene encoding a visual system-specific G alpha molecule. Neuron.

[50] Parks S., Wieschaus E. (1991). The *Drosophila* gastrulation gene concertina encodes a Gα-like protein. Cell.

[51] Hull J.J., Kajigaya R., Imai K., Matsumoto S. (2007). The *Bombyx mori* sex pheromone biosynthetic pathway is not mediated by cAMP. J. Insect Physiol..

[52] Hull J.J., Lee J.M., Matsumoto S. (2010). Gqalpha-linked phospholipase Cbeta1 and phospholipase Cgamma are essential components of the pheromone biosynthesis activating neuropeptide (PBAN) signal transduction cascade. Insect Mol. Biol..

[53] Horgan A.M., Lagrange M.T., Copenhaver P.F. (1995). A developmental role for the heterotrimeric G protein Go alpha in a migratory population of embryonic neurons. Dev. Biol..

[54] Raming K., Krieger J., Breer H. (1990). Molecular cloning, sequence and expression of cDNA encoding a G 0-protein from insect. Cell. Signal..

[55] Kang G.-J., Gong Z.-J., Cheng J.-A., Zhu Z.-R., Mao C.-G. (2011). Cloning and expression analysis of a G-protein α subunit-Gαo in the rice water weevil *Lissorhoptrus oryzophilus* Kuschel. Arch. Insect Biochem. Physiol..

[56] Qiao Q., Li H.-C., Yuan G.-H., Guo X.-R., Luo M.-H. (2008). Gene cloning and expression analysis of G protein αq subunit from *Helicoverpa assulta* (Guenée). Agric. Sci. China.

[57] Tu H., Qin Y. (2014). Cloning and expression analysis of G-protein Gαq subunit and Gβ1 subunit from *Bemisia tabaci* Gennadius (Homoptera: Aleyrodidae). Arch. Insect Biochem. Physiol..

[58] Ewen-Campen B., Jones T.E.M., Extavour C.G. (2013). Evidence against a germ plasm in the milkweed bug *Oncopeltus fasciatus*, a hemimetabolous insect. Biol. Open.

[59] Debolt J.W. (1982). Meridic diet for rearing successive generations of *Lygus hesperus*. Ann. Entomol. Soc. Am..

[60] Patana R. (1982). Disposable diet packet for feeding and oviposition of *Lygus hesperus* (Hemiptera: Miridae). J. Econ. Entomol..

[61] Brent C.S., Hull J.J. (2014). Characterization of male-derived factors inhibiting female sexual receptivity in *Lygus hesperus*. J. Insect Physiol..

[62] Strathmann M., Wilkie T.M., Simon M.I. (1989). Diversity of the G-protein family: Sequences from five additional alpha subunits in the mouse. Proc. Natl. Acad. Sci. USA.

[63] Knight P.J.K., Grigliatti T.A. (2004). Diversity of G proteins in Lepidopteran cell lines: Partial sequences of six G protein alpha subunits. Arch. Insect Biochem. Physiol..

[64] Ren J., Wen L., Gao X., Jin C., Xue Y., Yao X. (2008). CSS-Palm 2.0: An updated software for palmitoylation sites prediction. Protein Eng. Design Sel..

[65] Edgar R.C. (2004). MUSCLE: A multiple sequence alignment method with reduced time and space complexity. BMC Bioinform..

[66] Edgar R.C. (2004). MUSCLE: Multiple sequence alignment with high accuracy and high throughput. Nucl. Acids Res..

[67] Tamura K., Stecher G., Peterson D., Filipski A., Kumar S. (2013). MEGA6: Molecular evolutionary genetics analysis version 6.0. Mol. Biol. Evol..

[68] Jones D.T., Taylor W.R., Thornton J.M. (1992). The rapid generation of mutation data matrices from protein sequences. Comput. Appl. Biosci..

[69] de Sousa S.M., Hoveland L.L., Yarfitz S., Hurley J.B. (1989). The *Drosophila* Go alpha-like G protein gene produces multiple transcripts and is expressed in the nervous system and in ovaries. J. Biol. Chem..

[70] Ratnaparkhi A., Banerjee S., Hasan G. (2002). Altered levels of Gq activity modulate axonal pathfinding in *Drosophila*. J. Neurosci..

[71] Graveley B.R., Brooks A.N., Carlson J.W., Duff M.O., Landolin J.M., Yang L., Artieri C.G., van Baren M.J., Boley N., Booth B.W. (2011). The developmental transcriptome of *Drosophila melanogaster*. Nature.

[72] Sze S.-H., Dunham J.P., Carey B., Chang P.L., Li F., Edman R.M., Fjeldsted C., Scott M.J., Nuzhdin S.V., Tarone A.M. (2012). A *de novo* transcriptome assembly of *Lucilia sericata* (Diptera: Calliphoridae) with predicted alternative splices, single nucleotide polymorphisms and transcript expression estimates. Insect Mol. Biol..

[73] Venables J.P., Tazi J., Juge F. (2011). Regulated functional alternative splicing in *Drosophila*. Nucl. Acids Res..

[74] Thomas T.C., Schmidt C.J., Neer E.J. (1993). G-protein alpha o subunit: Mutation of conserved cysteines identifies a subunit contact surface and alters GDP affinity. Proc. Natl. Acad. Sci. USA.

[75] Posner B.A., Mixon M.B., Wall M.A., Sprang S.R., Gilman A.G. (1998). The A326S mutant of Gialpha1 as an approximation of the receptor-bound state. J. Biol. Chem..

[76] Marrari Y., Crouthamel M., Irannejad R., Wedegaertner P.B. (2007). Assembly and trafficking of heterotrimeric G proteins. Biochemistry.

[77] Scott K., Becker A., Sun Y., Hardy R., Zuker C. (1995). Gq alpha protein function *in vivo*: Genetic dissection of its role in photoreceptor cell physiology. Neuron.

[78] Hardie R.C., Martin F., Cochrane G.W., Juusola M., Georgiev P., Raghu P. (2002). Molecular basis of amplification in *Drosophila* phototransduction: Roles for G protein, phospholipase C, and diacylglycerol kinase. Neuron.

[79] Sunahara R.K., Taussig R. (2002). Isoforms of mammalian adenylyl cyclase: Multiplicities of signaling. Mol. Interv..

[80] Hewavitharana T., Wedegaertner P.B. (2012). Non-canonical signaling and localizations of heterotrimeric G proteins. Cell. Signal..

[81] Brabet P., Dumuis A., Sebben M., Pantaloni C., Bockaert J., Homburger V. (1988). Immunocytochemical localization of the guanine nucleotide-binding protein Go in primary cultures of neuronal and glial cells. J. Neurosci..

[82] Gabrion J., Brabet P., Nguyen Than Dao B., Homburger V., Dumuis A., Sebben M., Rouot B., Bockaert J. (1989). Ultrastructural localization of the GTP-binding protein Go in neurons. Cell. Signal..

[83] Wolfgang W.J., Quan F., Goldsmith P., Unson C., Spiegel A., Forte M. (1990). Immunolocalization of G protein alpha-subunits in the Drosophila CNS. J. Neuorsci..

